# Observational quality control study: insourcing multi-PCR-impact on the use of anti-infectives for patients with pleocytosis

**DOI:** 10.1186/s42466-025-00398-9

**Published:** 2025-06-20

**Authors:** Jörg Tebben, Bianca Wiebalck, Holger Schmidt

**Affiliations:** 1 Neurologie, Elbe Kliniken, Bremervörder Strasse 111, 21682 Stade, Lower Saxony Germany; 2 RSU Branch Stade, Rīga Stradiņš University, RSU, 16 Dzirciema Street, 1007 Riga, Latvia; 3 Neurologie, University Medical Center Göttingen, Robert-Koch-Str. 40, 37075 Göttingen, Lower Saxony Germany

**Keywords:** CSF-pleocytosis, ME-PCR, Laboratory inhousing, Laboratory outsourcing, inflammatory CNS process, meningoencephalitis, Multi ME PCR

## Abstract

**Background:**

An analysis of the cerebrospinal fluid (CSF) is essential for diagnosis of meningitis, headache, disturbance of conscience, cranial nerves or autoimmune-related conditions of the CNS. The initial treatment of pleocytosis usually consists of both antiviral therapy and antibiotics until laboratory results enable a more specific approach. Therefore, it is crucial to rapidly and accurately detect pathogens.

**Methods:**

In this observatory, monocentric study of quality management data, we studied insourcing of ME-PCR, CXCL 13, Antibody-specific Index (AI) for HSV, VZV (G$_{1}$) compared with outsourced laboratory measurements (G$_{0}$) and its benefit for the work-up. Before the implementation of these parameters, data from 150 patients were sampled, followed by 210 after the introduction of ME-PCR, CXCL 13 and AI. Data were collected, anonymized, and analysed afterwards. All were treated in hospital for suspected infections of the Central Nervous System (CNS). The length of hospital stay (LOS), intervals from lumbar puncture, the cumulative dose of anti-infective agents, length of treatment and the potential impact on patients’ safety parameters were examined.

**Results:**

The G$_{1}$-group showed a significant decrease of LOS (p<0.001), exposure to antiviral, and antibiotic agents decreased significantly (p < 0.001, each). Insourcing of ME-PCR and CXCL 13 shortened the time-span from admission to diagnosis in patients with suspected inflammatory CNS disease from 13.6 (6.6) to 9.7 (6.7) days in mean (SD).

**Conclusion:**

The shortened average LOS after changing the diagnostic pathway increased direct costs for test kits. However, these costs were by far outweighed the economical benefit of being able to treat more patients in the same time. This analysis should be replicated in a different Medical Care System than the one in which this analysis has been calculated.

## Introduction

Smaller hospitals often do not provide the complete microbiological analytic work-up, and are often forced to send CSF and other specimens obtained from patients with suspected meningitis or meningoencephalitis (ME) to external laboratories. As those can collect samples from multiple sources they have a high throughput per parameter, reducing costs for analytic kits and reactants. Therefore, centralizing laboratory services can be economically beneficial.

From the treating neurologist’s point of view, some of the diagnostic and therapeutic decisions can already be made using basic CSF data as leukocyte count, lactate, Albumin-CSF to serum ratio (Q$_{Albumin}$) or culture. The differential diagnosis between viral ME due to Herpes virus infections requiring Acyclovir (ACV) treatment and e.g., enteroviral ME, which should not be treated with antiviral drugs, can be difficult. Such difficulties can lead to “calculated” ACV-treatment which usually prolongs LOS until the final laboratory results (often PCR and antibody-specific indices) are available. Also, distinguishing viral meningitis from neuroborreliosis (NB) can be challenging at times, when typical signs of NB or cutaneous symptoms are missing. CXCL 13 as a B-lymphocyte chemoattractant can be of considerable help in this clinical setting [45]. These were the reasons for us to accelerate the determination of this parameter as well. In order to handle these difficulties occurring at the start of the treatment of patients with pleocytosis, combination therapies targeting both Herpes viruses and most of the bacteria causing meningitis are recommended (see e.g. German guidelines or [34], e.g. Infectious Diseases Society of America [48]. Those empiric treatments are given until the final diagnosis can be determined with sufficient certainty.

Having identified the correct cause, the therapy is usually replaced by the most specific one. It often takes several days of detailed neurochemical and microbiological analysis before such decisions can be made.

If a distinctive cause still can’t be found by standard of care such as culture or molecular biology techniques, the decision to discontinue either the antiviral, the antibacterial or both agents can only be made on the basis of clinical impression, biochemical findings and probabilistic reasoning.

An example of an initial empiric antibiotic regimen used in Central Europe consists of a 3rd generation cephalosporine and Ampicillin combined with Acyclovir (ACV). These safety considerations result in a futile over-treatment for many ME-patients until their definite diagnosis is established. During public holidays or weekends, transport logistics offered by the centralized laboratories or by mail depending on fixed schedules (e.g. twice a day), can prolong this delay even more.

In addition, referral laboratories collect specimens until a certain amount of orders is reached for a common measurement, e.g. on a 96 well plate. But many more predictable and unpredictable factors may prolong the interval from sample collection to the final result depending on various individual hospital settings.

We should always be aware that slower diagnostic procedures increase patients’ exposure to potentially harmful medications for a longer period of time:

In an animal model, it could be demonstrated that the toxicity of ACV is both dose- and time-dependent [52]. Administering drugs at higher doses usually being the case in patients with ME in order to cross the blood-brain-barrier, represents a considerable risk factor for idiosyncratic drug-induced liver injury [27]. Although orally administered ACV does not seem to induce regularly renal injury [26], this is not the case for intravenous use being obligatory in ME: Renal injury occurs from 15.6% [1, 30] to 38.2% [5]. The risk for renal dysfunction is with 62.7% much higher [1] for which crystallization of the drug in renal tubuli is responsible [38]. Not surprisingly, elderly and dehydrated feverish patients are at special risk to develop complications. Anemia and thrombocytopenia have also been described (e.g. [19]). Clinically, even more challenging for the treating neurologist is the unwanted side effect of encephalopathy caused sometimes by ACV [41], easily misconceived as a primary encephalitic symptom. ACV and its metabolite 9-carboxymethoxymethylguanine (CMMG) both have found to be responsible for this side effect [50].

Also antibacterial agents that are often considered harmless can induce renal and hepatic side effects. Ceftriaxone (CRO) e.g., can crystallize in the renal tubuli [53] or can form bile sludge and even gallstones [23]. Similar to ACV, CRO may also cause agranulocytosis as described by [18]. Diarrhea caused by the disturbance of enteric bacteria can lead to severe dehydration, electrolyte imbalance and clinical signs that are also difficult to differentiate from ME symptoms. Cases of primary encephalopathy due to cephalosporines were reported, too [20, 54].

Therefore, switching to specific therapy might not only help cutting down the length of hospitalization (especially in patients with meningitis due to *Enteroviridae*), but can save patients from adverse and avoidable drug-induced side effects.

Several years ago, ME PCR panels were introduced. The initial fear that their sensitivity might be too low could be dispersed [39]. Though being very sensitive, the sensitivity of ME PCR has been reported slightly lower than with standard single PCR [10, 28]. Especially, the detection of *Cryptococcus neoformans* seems to be challenging for ME multi PCR-setups [7].

Multi-PCR arrays targeting 14 pathogens commonly causing CNS infections have become available. Although these methods are nearly as sensitive as standard PCRs (apart from *C. neoformans*) and easy to perform [22], 14 tests are performed invariable at a time, making the procedure expensive.

In pediatric patients with HSV-encephalitis, Van et al. showed a significant decrease in time until the diagnosis was made [49]. Since patients with suspected Herpes encephalitis are treated empirically, the time span from specimen collection to initiation of ACT treatment was not diminished in contrast to a shortened period to discontinuation when the HSVE-diagnosis could be falsified.

We oversaw the internal implementation of two laboratory parameters often used for the differentiation of ME, and performed a quality survey to monitor and balance the medical benefit against the economical burden associated with this modification of our clinical pathway. The hypothesis that ME-PCR and CXCL 13 yielded an earlier diagnosis and could shorten the LOS leading had to be proven.

## Methods

### Setting

This monocentric study was performed in a hospital caring for approximately 4000 adult neurological in-hospital patients p.a.. The hospital is supplied with a computerized clinical data system providing an archive with both electronic and (few) hand-written forms. In 2018, we initiated an internal implementation project in order to increase the diagnostic speed of patients with suspected meningitis or meningoencephalitis. PCR analysis (mostly performed only for HSV and VZV), AI for Borrelia-IgM /-IgG, HSV-IgG, VZV-IgG, and CXCL 13 which had hitherto been sent to various external German laboratories, could then be measured in our local neurochemistry laboratory. PCR samples were sent by courier, CXCL 13 was sent by mail.

In 2018, there was an overlap of internal and external analysis of parameters mention above.

From 28.2.2019 to 20.1.2021 patients were included in the study, and CXCl 13 and ME-PCR were measured in-house without a,elevant transportation-induced delay. PCR diagnostics were performed with BioFire®Menigoencephalitis (ME) Panel on the FilmArray 2.0 system®(BioMerieux, which had been shown earlier to be comparable with the MALDI-TOF technique [37]. This panel tests for genomes from *Escherichia coli K1, Haemophilus influenzae, Listeria monocytogenes, Neisseria meningitidis, Streptococcus agalactiae, Streptococcus pneumoniae*, Cytomegalovirus (CMV), enterovirus, herpes simplex virus (HSV)-1, HSV-2, human Herpesvirus 6 (HHV-6), human parechovirus, varicella zoster virus (VZV) and *Cryptococcus neoformans/gattii* [29]. CXCL 13 was also performed with any pleocytosis $\ge$ 4/$\mu$l, measured with a recomBead (Mikrogen Neuried, Germany) multiplex MAGPIX®system (Luminex Corp., Austin, TX, USA). A cut-off value $\ge$ 250 pg/ml were used to be suggestive for Neuroborreliosis. Antibody specific CSF / serum indices for Herpes simplex and Varicella zoster viruses were determined semi-automatically using ELISA systems (Immunomat®, VirionSerion, Wuerzburg, Germany).

This implementation process was scientifically monitored to determine whether the increased workload and associated hospital costs were justified, particularly with regard to patients’ length of hospital stay, exposure to anti-infective drugs, and hepato-renal function.

### Study population

Patients treated from 2014 to 2017 served as a historical control group.

So this study was both retrospective (group 0, G$_{0}$) and prospective (group 1, G$_{1}$) one. In both groups, we included patients with suspected meningitis or meningoencephalitis (ME).

PCR tests were performed whenever the leukocyte count > 4/$\mu$l. Exceptionally, a PCR could be indicated with CSF leukocytes of $\le$ 4/$\mu$l if an infection of the CNS was suspected clinically (e.g. with meningism and fever without other proven reasons, with high systemical inflammatory parameters or when imaging was suspicious for meningoencephalitis).

PCR tests with CSF leukocyte counts $\le$ 4/$\mu$l were done in five patients of group 0, and in six of group 1. Patients were grouped according to their diagnosis into these classes: 1 VZV,2 HSV, 3 Enterovirus, 4 other Virus, 5 probable viral Meningitis, 6 Neuroborreliosis (NB), 7 *Str. pneumoniae,* (SP), 8 *Listeria monocytogenes* (LM), 9 *Neisseria meningitidis* (NM), 10 *Haemophilus influenzae* (HiB), 11 other bacterial origin, 12 probable bacterial meningitis, 13 autoimmune CNS inflammation, 14 Stroke with inflammatory CSF reaction, 15 unknown.

In order to allow group comparisons, these groups were further simplified into the following categories: 0 viral, 1 bacterial, 2 Borrelia, 3 autoimmune, and 4 other or unknown.

#### Parameters

Laboratory parameters incorporated into the analysis were CSF-Leukocytes [$\mu$l], CSF-Lactate [mmol/l], the CSF/serum ratio of Albumin [x 1E3] (Q$_{Albumin}$), CSF-TPHA, AI for HSV, VZV-AI, Borrelia-IgG AI, Borrelia IgM-AI, CXCL 13 [pg/ml], result of PCRs, and CSF culture results.

Biochemically, creatinine at admission, at the last available creatinine, the creatinine increase from admission to the day of discharge (DOD), urea at admission and DOD, the Urea-increase, gamma-glutamyl transferase (gGT) [U/l] at DOA and DOD, Alanine aminotransferase (ALT) [U/l] at DOA/DOD, Blood Leukocytes (BLC) [$\mu$l] at DOA/DOD, and Thrombocytes [$\mu$l] at DOA/DOD were determined.

In addition to the length of hospital stay (LOS), the intervals from DOA to incoming results were taken.

The cumulative dose of antiviral and antibiotic agents, respectively, was calculated for each patient.

Because of the variety of anti-infectives used we aimed to build an adjusted cumulated dose as referred to a similar hypothetical dose that was given when ACT or CRO had been administered. When Valacyclovir was given, e.g., with the recommended daily dose (3x1g), it was estimated to be equivalent to ACV 3x750mg/d.

The duration of antibacterial and antiviral therapies was recorded.

### Treatments

Most of the patients initially received an calculated treatment consisting of ACV 10 mg/kg i.v. three times daily, in addition to CRO 2 g i.v. twice daily ± Ampicillin (AMP) 3 g four times a day. Some of the patients claimed premature discharge from the hospital before antiviral or respective antibiotic therapy was completed. These patients were treated with a sequential oral therapy scheme, mainly Valacyclovir when viral aetiology was suspected. We calculated the total dose of antibiotic and antiviral agents. The total amount of applied drugs is especially important for the liver and renal function. However, to enable better comparison between individual therapies, we standardized dosing by calculating equivalent doses for all patients who received antiviral (AVX) or antibiotic(ABX) agents other than ACV or CRO. For example, patients who received oral Valacyclovir (3 × 1 g/day) as squential therapy were converted to the equivalent of 3 × 750 mg intravenous Acyclovir.

### Statistics

#### Procedures

After data collection, personal data were erased from the spreadsheet (BW) and the dataset was handed over to anonymized data analysis. Statistical calculations and tables were performed with JASP, version 0.18.3.0 [25] which provides a user interface for R, version 4.2.1 [40] as statistical engine. Graphs were produced with Jamovi, version 2.5.6.0 [47], also using R as its core engine.

In order to estimate the needed group sizes, power calculations were performed with G-Power 3.1.9.6 [16] with a two-tailed* t* test with a given significance level of 0.05 and a power of 90%.

Referring to data from a study with paediatric patients and a similar issue [21] we estimated a LOS of 10 ± 2 days for the retrospective group and of 7 ± 2 days for the prospective meningitis group with PCR- and CXCL 13 diagnostics. According to this assumption, the effect size of the change in work-up was 1.5, resulting in a minimal group size of N = 11.

Referring to a database analysis from previous years, we expected the frequency of patients with CNS inflammations to be approximately 90 per year.

Welch’s* t* test for independent samples was applied to find group differences due to its better performance in case of unequal variance [11]. As our group size was larger than N = 50 for both G$_{0}$ and G$_{1}$, we used parametrical test statistics regardless of their distribution [33, 44].

We used linear regression analysis with a cut-off* p* value of < 0.05 from the Pearson’s correlation test as statistically significant. In this case, coefficients of > 0.5 or < $-$0.5 were considered to be clinically relevant [9].

## Results

### Patients

Demographic properties of both groups were similar: G$_{0}$ (outsourced diagnostics): N = 150; f/m: 73/77), and G$_{1}$ inhouse ME-PCR / CXCL 13: N = 210; f/m: 101/109, $\chi ^{2}$ p = 0.9. G$_{0}$ were consecutively treated patients from 2013 to 2017, G$_{1}$ from 2019 to 2021. During 2018, the new procedures were introduced. In 2018, both clinical pathways for the determination of PCR and CXCL 13 were used simultaneously. Therefore, this year was excluded from the analysis.

The age of both groups at admission did not differ: 57.9 $_{G0}$ ± 20.2 and 58.0$_{G1}$ ± 19.6 years (*p* = 1.0). Similarly, no significant differences were found in CSF lactate, Q$_{Albumin}$, renal and hepatic function tests, thrombocyte counts, or blood leukocyte counts, as determined by* t* tests (Table 1).

**Table 1:** Demographic data, groupwise

Parameters at DOA*	Group	Valid	Missing	Mean	Std. Dev.	25th perc.	50th perc.	75th perc.
Age* [yrs]	0	150	0	57.9	20.2	42.0	62.0	75.8
	1	210	0	58.0	19.6	43.0	59.0	74.8
CSF Leukos* [/$\mu$l]	0	150	0	222.3	514.2	22.0	64.5	148.8
	1	210	0	159.8	688.4	8.0	17.5	74.0
Q$_{Albumin}$*	0	150	0	14.0	15.4	6.5	10.4	15.9
	1	210	0	15.6	35.4	5.9	8.1	13.2
CSF Lactate* [mmol/l]	0	150	0	2.3	1.0	1.7	2.1	2.7
	1	210	0	2.6	2.6	1.6	1.9	2.4
Creatinine* [mg/dl]	0	150	0	1.0	0.5	0.8	0.9	1.1
	1	210	0	1.0	0.8	0.8	0.9	1.1
Urea* [mg/dl]	0	148	2	15.4	9.2	10.8	13.8	16.9
	1	203	7	17.0	12.8	11.1	14.1	19.3
$\gamma$GT* [U/l]	0	146	4	41.2	57.5	17.0	25.0	45.0
	1	192	18	45.5	62.5	18.0	27.0	44.8
ALT* [U/l]	0	146	4	36.0	96.9	15.0	22.0	31.8
	1	193	17	40.6	95.1	15.0	22.0	37.0
Blood Leukos* [/$\mu$l]	0	150	0	9.4	4.1	7.0	8.5	10.9
	1	210	0	9.3	4.5	6.7	8.4	10.0
Thr* [/$\mu$l]	0	150	0	250.4	84.1	198.5	247.0	301.8
	1	210	0	254.9	79.4	205.0	249.0	299.8

Group	Diagnosis	Frequency	Percent	Valid percent	Cumulative percent
0	0	103	68.7	68.7	68.7
	1	15	10.0	10.0	78.7
	2	19	12.7	12.7	91.3
	3	3	2.0	2.0	93.3
	4	10	6.7	6.7	100.0
	Missing	0	0.0		
	Total	150	100.0		
1	0	93	44.3	44.3	44.3
	1	12	5.7	5.7	50.0
	2	17	8.1	8.1	58.1
	3	38	18.1	18.1	76.2
	4	50	23.8	23.8	100.0
	Missing	0	0.0		
	Total	210	100.0		

*: Welch’s test not significant; DOA: at date of admission, Group: 0= before insourcing , 1= after insourcing, CSF: cerebrospinal fluid, Q$_{Albumin}$: CSF to serum ratio of Albumin, $\gamma$GT: $\gamma$-glutamyl transferase, ALT: alanin amino transferase

### Time intervals

**Fig. 1:**
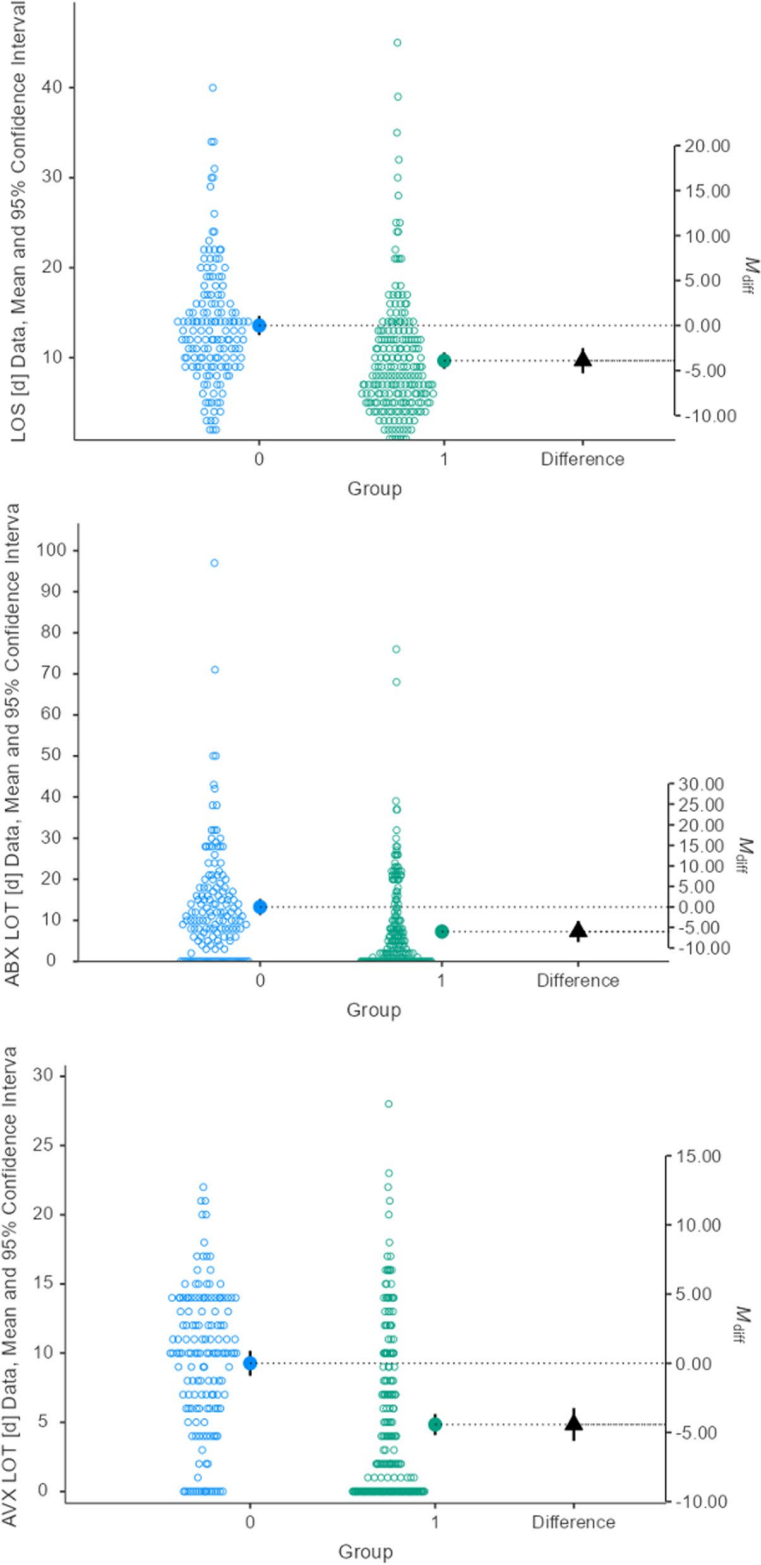
Effect of introducing in-house laboratory diagnostics. LOS: Length of hospital stay, LOT: Length of therapy, ABX: antibiotic agent, ABV: antiviral Agent

Due to in-housing diagnostics a significantly shortened time interval from lumbar puncture to laboratory result was obtained (Table 2). Not only these differences were significantly lower but the days of antiviral and antibiotic therapy decreased (Fig. 1).

The introduction of CXCL 13, however, did not influence the LOS (9.24 ± 7.83 d$_{G0}$ vs. 5.81 ± 6.86 d$_{G1}$) nor the total antibiotic dose (39.26 ± 57.0 g$_{G0}$ vs. 17.1 ± 27.71 g$_{G1}$), t-test p = 0.08 and p = 0.09, respectively in patients with neuroborreliosis.

In Table 3, the correlations of different time intervals with LOT and LOS are given: LOTs and LOS correlated significantly with most of them, the Pearson’s r of LOS and LOTs, however, did not reach the level of 0.5.

**Table 2:** Comparison of time-dependent variables

Parameter	Group	N	Mean	SD	CV
LOS* [d]	0	150	13.6	6.6	0.5
	1	210	9.7	6.7	0.7
Span CSF 1 to HSV-AI* [d]	0	72	6.4	4.7	0.7
	1	204	1.6	1.1	0.7
Span CSF 1 to VZV-AI* [d]	0	112	6.2	6.7	1.1
	1	205	1.6	1.1	0.7
Span CSF 1 to Borrelia AI* [d]	0	109	4.9	3.1	0.6
	1	210	1.5	1.1	0.7
Span CSF 1 to CXCL 13* [d]	0	51	16.4	9.5	0.6
	1	201	1.6	1.1	0.7
Span CSF 1 to HSV/VZV-PCR* [d]	0	150	7.6	7.2	0.9
	1	210	1.2	0.9	0.8
ABX LOT* [d]	0	150	13.3	13.7	1.0
	1	210	7.3	11.0	1.5
AVX LOT* [d]	0	150	9.3	5.2	0.6
	1	210	4.8	5.9	1.2

SD: Standard deviation, CV: Coefficient of variation, d: day(s). *: Welch’s test p < 0.001, LOS: Length of hospital stay, CSF: cerebrospinal fluid, AI: Antibody specific index, HSV: Herpes simplex virus, VZV: Varicella zoster virus, Borrelia AI: IgG- or IgM-AI of Antibodies against *Borrelia burgdorferi sensu latu*, ABX: antibiotic medication, LOT: length of therapy, AVX: antiviral medication

**Table 3:** Pearson’s correlations

Variables’ r	LOS	ABX LOT	CROU	AVX LOT	ACVU	IntHSV-AI	IntVZV-AI	IntBB-AI	IntCXCL13
1. LOS	–								
2. ABX LOT	0.5	–							
3. ABX CROU	0.2	0.7	–						
4. AVX LOT	0.3	0.2	0.2	–					
5. AVX ACVU	0.1	0.1	0.2	0.7	–				
6. Int. HSV-AI	0.1	0.1	0.1	0.2	0.1	–			
7. Int. VZV-AI	0.2	0.1	0.1	0.3	0.1	0.6	–		
8. Int. BB-IgM/G AI	0.2	0.2	0.2	0.3	0.1	0.7	0.8	–	
9. Int. CXCL 13	0.3	0.2	0.2	0.3	0.2	0.5	0.7	0.7	–
10. Int PCR	0.3	0.2	0.1	0.3	0.1	0.7	0.9	0.8	0.7

LOS: length of hospital stay, ABX: antibiotics, LOT: length of therapy, CROU: Ceftriaxone equivalence dose, AVX: antiviral therapy, ACVU: Acyclovir equivalence dose; IntXXX: Interval from lumbar puncture to XX-result, HSV: Herpes simplex virus, VZV: Varicella zoster virus, BB: *Borrelia burgdorferi*

### Laboratory parameters

The initial laboratory parameters associated with inflammatory burden (CSF and blood leukocytes, Q$_{Albumin}$, CSF lactate, CSF CXCL 13) did not differ between G$_{0}$ and G$_{1}$, all Welch-test p-values exceeded the level of p > 0.05 .

Neither one of the kidney nor of the liver function tests showed a significant difference at the time of admission.

Likewise, the thrombocyte counts of G$_{0}$ and G$_{1}$ were comparable (*p* < 0.05). None of the collected samples were positive for the CSF Lues test (TPHA).

PCR positivity rates were 18.7 and 18.6% for G$_{0}$ and G$_{1}$ ($\chi ^{2}$ p = 0.73).

The shorter duration of the treatments and decreased exposure to ABX and AVX did neither correlate with better liver or kidney function tests, nor were leukocyte and thrombocyte counts higher in the G$_{1}$ group due to the shorter treatment (all Welch-tests* p* > 0.05).

We transformed the data to nominal data (parameter increased from DOA to DOD = 1 or not increased during this period = 0) and calculated a $\chi ^{2}$ statistics for the four field tables with G$_{0}$ and G$_{1}$ as grouping variables. None of the $\chi ^{2}$ statistics revealed a significant difference (*p* values all > 0.05).

**Fig. 2:**
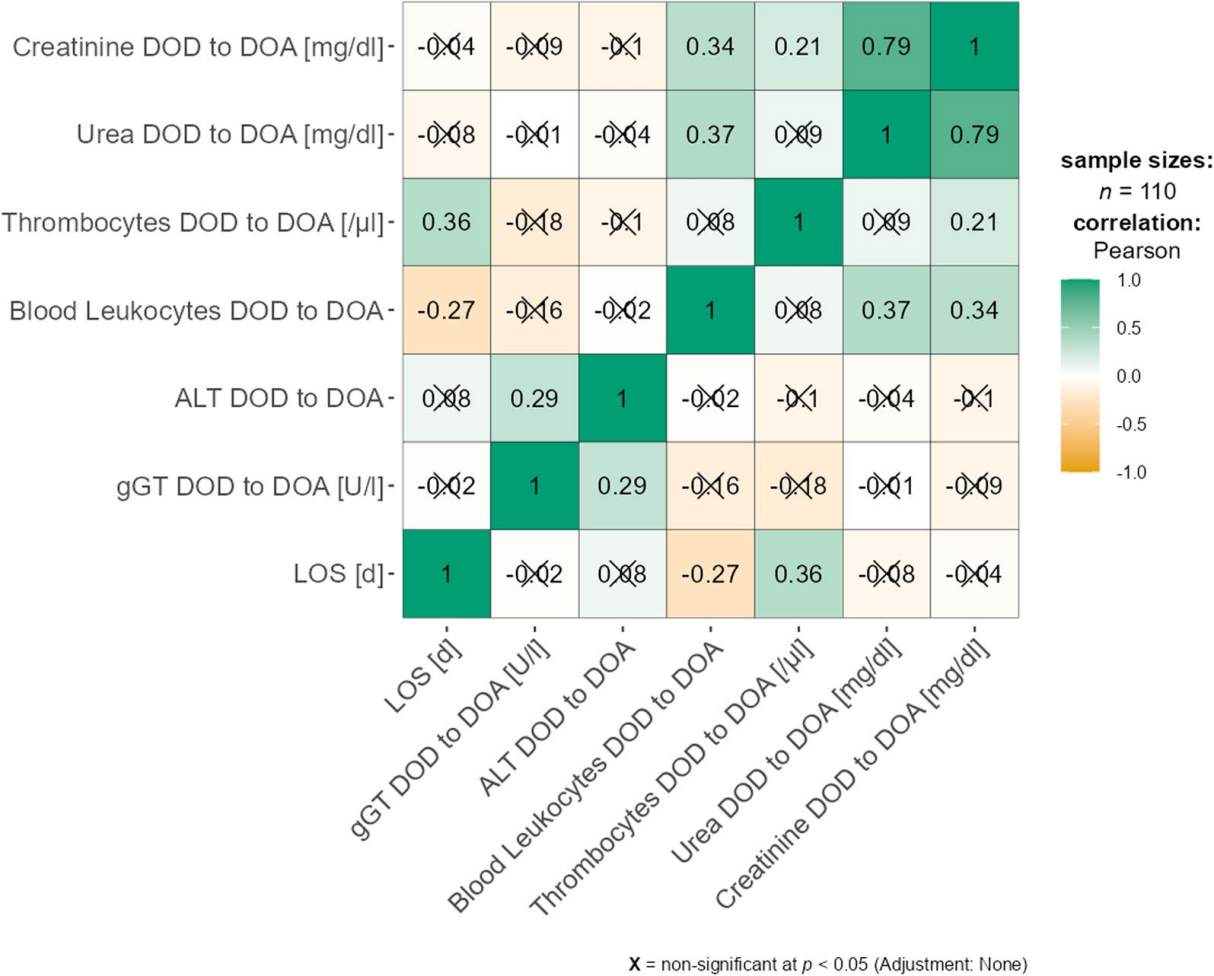
Heatmap for Pearsons's correlations with correlation coefficients and significance. DOD to DOA: Difference between day of Admission and day of discharge for the respective Parameter. LOS: Length of stay

Likewise, in the correlation matrix, neither total dose of ABX nor those of AVX pointed to a clinically relevant relation with the laboratory parameters mentioned above (correlation coefficients all > 0.5), see Fig. 2.

The duration of hospital stay was statistically significantly, and positively correlated with the duration of the dynamics of the laboratory parameters under investigation. However, the Pearson regression coefficients for all the tested parameters was < 0.5, therefore probably these correlations will be clinically irrelevant.

### Treatment duration and applied doses

The implementation of in-house diagnostics for the parameters mentioned above reduced the treatment duration with antiviral and antibiotic agents significantly. This corresponded to the significant lower drug exposition in G$_{1}$ compared to G$_{0}$. In mean, for G$_{1}$-patients the hospital stay was 3.9 days shorter than for those whose diagnostic workup was carried out externally. This difference was statistically significant (*p* < 0.001). Likewise, the duration of antiviral (9.3 vs. 4.8 days) and of antibiotic therapy (13.3 vs. 7.3 days) were significantly shorter (both* p* < 0.001).

The applied doses of ABX (70.8$_{G_{0}}$ vs. 24.2$_{G_{1}}$ g) and AVX (19.4$_{G_{0}}$ vs. 11.3$_{G_{1}}$ g), both* p* < 0.001 corresponded to the findings for duration of therapy.

## Discussion

Whenever established clinical pathways are changed, the responsible laboratory and clinical physicians should weigh the real world benefit for the patients against economic rationale. For the clinical scenario of meningoencephalitis, we compared the insourcing of cost-intensive but pivotal laboratory parameters with the established pathway to send the specimens as fast as possible to bigger laboratories specialized for neurochemistry which had been previously established long before.

The introduction of such panel diagnostics have been widely discussed - with different conclusions (see e.g. [4]). In addition to the Biofire®ME-Panel, other multi-PCR panels have been invented and made commercially available.

Our results showed a significant benefit for the patients through the shortened time span of empirical multi-modal treatment and LOS. The reduction in hospital stay was achieved mainly by identifying patients with benign viral meningitis who could be discharged early because no specific therapy is needed.

In addition, the accelerated diagnosis of neuroborreliosis (NB) supported by CXCL 13 significantly attributed to the decrease of LOS as NB can sometimes be difficult to differentiate from viral meningitis.

In our hospital we saved nearly four days of LOS. Although we were unable to prove a definite and meaningful clinical benefit for renal or hepatic function, four days correspond approximately to the duration of an average hospital treatment in German neurological departments. This being said, every patient with pleocytosis diagnosed earlier according to our pathway due to insourcing allowed us to discharge this patient earlier and treat one additional “average patient” over four days.

Provided that this “average patient” could again be treated within these four days, the costs of additional diagnostic measures for pleocytosis encompass less than 10% of the additional earnings that a hospital in the German health care system could achieve. This shortening effect of ME-PCR on LOS appeared to be stable and reproducible in various studies. For meta-analysis, please refer to [24].

Performing the laboratory diagnostics in huge cross-regional laboratories certainly has its advantages, too. By enabling en-gros purchase of reagents it allows considerable savings. As it does not bind laboratory work-forces in the hospital, outsourcing can have comfortable aspects. However, the costumer (hospitals and their patients) have to pay for this gain of comfort with a complete loss of control on the speed and priorization of the laboratory process. Moreover, Soucek et al. could show for the multi-PCR that it can even cause savings when competing with an established pathway for pleocytosis that already included single PCR [46]. This finding is underpinned by the considerable treatment costs for meningoencephalitis: These costs accumulate in mean ± SD to 15572 ± 27168 $[3], so each hospital day that could be saved is important.

The limitation of our study is certainly its monocentric character. Therefore, neither a transfer of our results to other infrastructural hospital surroundings nor conclusions for other health systems should be drawn. Our hospital had previously established a basic set of parameters that enabled us to already diagnose some of the ME patients very early, e.g. by culture, stain or microscopy before without implementation of AI’s, PCR and CXCL 13.

Therefore, this setting might have led to an underestimation of the effect for hospitals that have to send all of their CSF to external laboratories.

On the other hand, compared to a university setting with a multitude of available different PCRs, the implementation of ME panels was not able to decrease LOS or LOT [12].

In another hospital that already offered vast choice of PCR diagnostics, however, the time saving until the exact diagnosis still was > 17 h [49]. In such hospital structures with a high throughput, one has to discuss if single-PCRs were a possible alternative to Poly-PCRs [7, 10]. A report from a hospital with a similar structure as the investigators’ described according to our observation that sending PCRs away caused significantly higher LOS [13].

In our geographical region, ME caused by fungi is a very rare event. So safety concerns of lower sensitivity for *Cryptococcus neoformans* detection did not play a relevant role in our hospital. In regions with a higher prevalence of fungal meningitis [17] (e.g. due to a high number of HIV positive patients) insourcing of BIOFIRE ME Panel could be less advantageous [8] when extra PCRs had to be ordered routineously for fungi to provide a maximal sensitivity [32]. Certainly, adding this multi-PCR is better than culture and microscopy alone [43]. The impact of additional diagnostic tools certainly depends extensively on the pre-existing setting: In the study of Wooton et al. the finding of formerly not detected pathogens increased the diagnostic certainty [51] and was significantly better than microscopy and culture alone [35]. Apart from the speed of diagnosis and the saving of antibiotics the proper benefit for the individual patient (who had probably already been treated efficiently by applying an empiric scheme, too) remains uncertain.

Some of the PCRs included in the ME-PCR panel were not very useful in our region and in the treated adult population, e.g. *E.coli* or Cytomegalovirus (CMV) have a low local prevalence in our patients with pleocytosis. Since the PCR panel could not be modified individually at the time of this study, from an economic point of view we automatically ordered too many and futile parameters.

On the other hand, for producers of such multi-PCR assays, other age and patient groups have to be taken into account (e.g. infants [2, 6]), and official regulations will make it impossible to provide individually adapted solutions due to the usual requirements for quality controls of diagnostic tools.

While in our situation the ME-PCR panel might rather provide too many genomes and may lead to a considerable overuse of diagnostic (and financial) means, for other geographic regions important genomes are missing in the panel (e.g. for tuberculosis) [31].

We are aware that the COVID-19 pandemic could have affected the LOS. In order to control for this factor, we calculated an in-group comparisons of LOS in G$_{0}$ of those patients who had received the ME-PCR and who were included before or after the first Corona patient in Lower Saxony was diagnosed (5.2.2020). This difference was not significant (LOS before Corona: 10.0 ± 6.05 vs. LOS within Corona-period 9.27 ± 7.42 days, t-test p = 0.421). Because of the small numbers of patients per diagnostic category, we could not evaluate this influence the potential effect of Corona on the frequency of patients in the diagnostic subgroups of G$_{0}$.

For our area, the simultaneous introduction of CXCL 13 as a marker for lymphocyte activation being very useful to differentiate neuroborreliosis from viral meningitis was as important as the ME-PCR panel in our clinical practice even though the local guidelines do not estimate this parameter to be crucial, yet [42].

The introduction of CXCL 13 did not influence LOS or the total antibiotic dose in patients with neuroborreliosis.

Certainly, the hypothetically isolated introduction of ME-PCR with already established AI- and CXCL 13 measurement capabilities would have decreased the differences between the two clinical pathways. In such situations, the effect on diagnostic speed of viral causes of pleocytosis seems to be more marked than that for bacterial ones [14, 36].

## Conclusion

Our observational quality management study could not prove to reduce side effects from prolonged calculated antibacterial and antiviral therapies. However, we reckoned that the significantly shortened exposure to potentially harmful medications coupled with a considerable economic benefit for the hospital was reason enough to stick with the extended in-house procedure of pleocytosis. As especially a positive PCR for Enteroviridae is both important for the patient and - yielding a discharge-signal - for our department, the implementation of the Biofire®-ME PCR has led to a regular use in patients who display > 10 leukocytes$\mu$l and to a restricted use for those between 5 and 10 leukocytes/$\mu$l. As a result of our study, we implemented the parameters in a similar way as other hospitals [15].

## Limitations

- Pandemic Confounding: While controlled for statistically, hospital policy changes during the COVID-19 pandemic may still have influenced LOS.
- Small Subgroup Sizes: Diagnostic subgroup analyses (e.g., for specific pathogenes) were underpowered and inconclusive.
- Single-Center Design: Results may not generalize to other healthcare systems or larger hospital infrastructures.
- Sensitivity and Specificyty of tests: no head-to-head comparison of ME-PCR and AI was conducted, and the study acknowledges ME-PCR limitations (e.g., for Cryptococcus).
- Cost-effectiveness assessment: While economic arguments are made, no formal health economical analysis was performed.

1. Approval: Hereby, we confirm that our experimental protocol was approved by the local hospital research committee at the Forschungs- und Studienzentrum der Elbe Kliniken (FSE), Buxtehude (Reg. No. 096-EKS-NEU).
2. Accordance: The methods we used were carried out according the relevant guidelines and regulations of our hospital and country.

## Data Availability

A CSV table will be provided in the supplements.

## Abbreviations

CSF: Cerebrospinal fluid
AI: Antibody specific Index
CNS: Central Nervous System
ME-PCR: Meningoencephalitis-Polymerase Chain Reaction
CXCL 13: C-X-C motif chemokine 13
HSV: Herpes simplex virus
VZV: Varicella zoster virus
LOS: Length of stay
Q$_{Alb}$ / Q$_{Albumin}$: Albumin CSF/serum ratio
ACV: Acyclovir
NB: Neuroborreliosis
CRO: Ceftriaxone
IgG: Immunoglobulin G
HHV6: Human Herpesvirus 6
ELISA: Enzyme linked immunosorbent Assay
Str.: Steptococcus
DOD: Day of Discharge
gGT: $\gamma$glutamyl transferase
ALT: Alanine Aminotransferase
BLC: Blood Leukocytes
AMP: Ampicillin
gGT: Gamma-glutamyl
LOT: Length of Therapy
ABX: Antibiotic treatment
AVX: Antiviral treatment
SD: Standard deviation
CV: Coefficient of variation
CMV: Cytomegalo Virus
